# Cathepsin‐L Ameliorates Cardiac Hypertrophy Through Activation of the Autophagy–Lysosomal Dependent Protein Processing Pathways

**DOI:** 10.1161/JAHA.113.000191

**Published:** 2013-04-24

**Authors:** Mei Sun, Maral Ouzounian, Geoffrey de Couto, Manyin Chen, Ran Yan, Masahiro Fukuoka, Guohua Li, Mark Moon, Youan Liu, Anthony Gramolini, George J. Wells, Peter P. Liu

**Affiliations:** 1 Division of Cardiology, Heart and Stroke/Richard Lewar Centre of Excellence, University Health Network, Toronto General Hospital, 200 Elizabeth Street, Toronto, Ontario, Canada (M.S., M.O., G.C., M.C., M.F., G.L., M.M., Y.L., P.P.L.); 2 McMaster University Medical School, Hamilton, Ontario, Canada (R.Y.); 3 Department of Physiology, University of Toronto and University Health Network, Toronto, Ontario, Canada (A.G.); 4 Department of Epidemiology and Statistics, University of Ottawa Heart Institute, Ottawa, Ontario, Canada (G.J.W.); 5 University of Ottawa Heart Institute, Ottawa, Ontario, Canada (P.P.L.)

**Keywords:** autophagy, cathepsin‐L, lysosome, remodeling, ubiquitin‐protein

## Abstract

**Background:**

Autophagy is critical in the maintenance of cellular protein quality control, the final step of which involves the fusion of autophagosomes with lysosomes. Cathepsin‐L (CTSL) is a key member of the lysosomal protease family that is expressed in the murine and human heart, and it may play an important role in protein turnover. We hypothesized that CTSL is important in regulating protein processing in the heart, particularly under pathological stress.

**Methods and Results:**

Phenylephrine‐induced cardiac hypertrophy in vitro was more pronounced in CTSL‐deficient neonatal cardiomyocytes than in in controls. This was accompanied by a significant accumulation of autophagosomes, increased levels of ubiquitin‐conjugated protein, as well as impaired protein degradation and decreased cell viability. These effects were partially rescued with *CTSL1* replacement via adeno‐associated virus–mediated gene transfer. In the in vivo murine model of aortic banding (AB), a deficiency in CTSL markedly exacerbated cardiac hypertrophy, worsened cardiac function, and increased mortality. *Ctsl*^−/−^ AB mice demonstrated significantly decreased lysosomal activity and increased sarcomere‐associated protein aggregation. Homeostasis of the endoplasmic reticulum was also altered by CTSL deficiency, with increases in Bip and GRP94 proteins, accompanied by increased ubiquitin–proteasome system activity and higher levels of ubiquitinated proteins in response to AB. These changes ultimately led to a decrease in cellular ATP production, enhanced oxidative stress, and increased cellular apoptosis.

**Conclusions:**

Lysosomal CTSL attenuates cardiac hypertrophy and preserves cardiac function through facilitation of autophagy and proteasomal protein processing.

## Introduction

Cardiac myocytes are long‐lived postmitotic cells, and the regulation of cell size and the dynamic turnover of proteins are key mechanisms of adaptation to variations in workload and stress in the heart. Cellular pathways participating in cell growth, survival, and death are controlled by the steady‐state level of regulatory proteins and enzymes, which result from the balance between protein synthesis and degradation. The rate of protein turnover also modulates protein damage and quality control, which in turn maintains the functional integrity of the cell. If this system is compromised, impaired protein degradation and abnormal protein accumulation may result in not only cellular dysfunction but also organ failure including cardiomyopathy.

The majority of proteins in eukaryotic cells are degraded via 2 proteolytic systems: the autophagy–lysosomal system and the ubiquitin–proteasome system (UPS).^1^ In cardiac muscle, these 2 systems are coordinated to preserve a carefully regulated composition of proteins and organelles.^2–3^ Proteasomes are thought to be responsible for the highly selective degradation of cellular proteins under basal metabolic conditions. Proteasome pathway targets soluble, short‐lived proteins by ubiquintination,^4–6^ followed by hydrolysis via the proteasome. In contrast, the autophagy–lysosomal pathway is responsible for the degradation of long‐lived bulky cytosolic proteins and organelles altered under conditions of stress (macroautophagy).^7–8^ This is also accompanied by lysosomal sequestration and degradation of additional specific cytosolic proteins (microautophagy and chaperone‐mediated autophagy).^9^

Impaired or dysregulated protein degradation in the heart has recently been recognized as a potential major contributor to cardiac disease. While the UPS and autophagy pathways have largely been investigated separately,^10–11^ there likely are significant cross‐talks and coordinations between these systems to optimize protein quality control. Cathepsin‐L (CTSL) is a key member of the lysosomal protease family in the heart. Mice lacking CTSL spontaneously developed heart disease at 1 year of age that resembled many features of human dilated cardiomyopathy.^12–13^ We have previous shown that deficiency of lysosomal enzyme CTSL in the setting of ischemic injury caused exaggerated activation of UPS.^14^ We believe that CTSL, an important lysosomal protein–processing enzyme, may play a key role in maintaining the lysosomal degradation response to stress. Cardiac hypertrophy, a critical hallmark of stress remodeling, involves progressive alterations of intracellular components including myofibrillar protein assembly.^15^ The final step of the autophagy–lysosomal pathway is the fusion of an autophagosome with a functioning lysosome, and an imbalance of protein homeostasis by dysfunction of this system may lead to pathological hypertrophy and dysfunction. It has been reported that cardiac myocyte–specific expression of CTSL in a CTSL‐deficient dilated cardiomyopathy mouse results in improved cardiac function.^16^ Although these observations suggested that changes in CTSL levels may cause altered cardiac function with stress, the importance of lysosomal proteases in cardiac protein turnover is still not well understood.

In the following sets of experiments, we examine how CTSL perturbation affects protein turnover and cardiac function in the setting of pressure‐overload hypertrophy.

## Methods

### Cell Culture

Cardiac myocytes were isolated from the ventricles of 1‐day‐old neonatal mice. Myocytes were dissociated with 0.15% trypsin in (in mmol/L) NaCl 137, KCl 5.4, MgSO_4_ 0.8, dextrose 5.6, KH_2_PO_4_ 0.4, Na_2_HPO_4_ · 7H_2_O, and HEPES 20.06 mmol/L, pH 7.4). Cells were differentially plated for 1 hour to remove fibroblasts and other contaminating cell types. The cells were then resuspended in DMEM/F12 medium supplemented with 10% fetal bovine serum, gentamicin (100 IU/mL), and bromodeoxyuridine. Myocytes were plated at a density of 2×10^5^ cells/mL, and serum was withdrawn after 24 hours. The cells were then treated for the following .

### Detection of CTSL Enzyme Activity

CTSL proteolytic activity was determined in tissue lysates (50 μg protein) by degradation of the fluoropeptide Z‐Phe‐Arg‐4‐methyl‐coumarin‐7‐amide (20 μmol/L; Bachem) in the presence of the CTSB‐specific inhibitor CA074 (1.5 μmol/L; Bachem) at a pH of 5.5. The release of 7‐amino‐4‐methyl‐coumarin was monitored continuously for 1 hour by spectrofluorometry at excitation and emission wavelengths of 360 and 460 nm.

### Western Blot Analysis

Cardiac myocytes or cardiac tissue samples were homogenized with lysis buffer and diluted 1:1 with 2× SDS sample buffer (Invitrogen Novex). An equal amount of protein (20 μg) was loaded onto each lane of an 8% to 16% Tris‐Glycin gel (Helixx). Proteins were separated by electrophoresis and transferred to a nitrocellulose membrane using an electroblotting apparatus (Invitrogen). Membranes were incubated with 5% BSA for 1 hour to decrease nonspecific binding. Samples were then incubated with the following primary antibodies overnight at 4°C: LC3 (MBL, Japan); ubiquitin and cytochrome c (Cell Signaling Technology); cathespin‐S (CTSS), cathespin‐D (CTSD), myosin, H‐cadherin, and Connexin43 (Abcam, Inc); atrial natriuretic factor (ANF), heat shock protein (Hsp)70, green fluorescent protein (Grp)94, and Grp78 (Stressgen, Bioreagents); Lamp‐1 (Santa Cruz Biochemistry, Inc.); and human CTSL1 (Lifespan Biosciences, Inc.). Samples were washed and incubated with peroxidase‐conjugated secondary antibody, and detected using the Amersham ECL detection kit.

### Immunofluorescent Staining of Myocytes

Cultured neonatal myocytes were washed briefly with PBS and fixed with 4% paraformaldehyde/PBS for 10 minutes at room temperature. The cells were then rinsed with PBS and incubated with 10% BSA, followed by overnight incubation with primary antibodies against CTSL (BD PharMingen Technical), α‐actinin (Sigma), Lamp‐1, LC3, and ubiquitin (P4D1; Cell Signaling Technology) at 4°C. The sections were then incubated with a matching fluorescent conjugated secondary antibody for 45 minutes at room temperature. Negative controls were performed for all immunologic staining by omitting the primary antibody.

### Reverse Transcription–Polymerase Chain Reaction

Total RNA was extracted from cultured myocytes/tissues. Reverse transcription–polymerase chain reaction (RT‐PCR) was performed with primers 5′‐ATGTGCCGGACCTTGGAAG‐3′ (forward) and 5′‐CCTCGGGTTAGCTGAGAGATCA‐3′ (reverse) for mouse beta‐myosin heavy chain (β‐MHC) and 5′‐CATGGCCTTCCGTGTTCCTA‐3′ (forward) and 5′‐CCTGCTTCACCACCTTCTTGAT‐3′ (reverse) for mouse GAPDH. With PCR buffers and Taq1 polymerase, the cycle parameters were as follows: denaturation at 95°C for 2 minutes, annealing at 60°C for 1 minute, and extension at 72°C for 2 minutes for 33 cycles with 5 minutes of final elongation at 75°C. Reaction mixture without template cDNA was used as a negative control.

### Myocyte Viability Assay

The 3‐[4,5‐dimethylthiazol‐2‐yl]2.5‐diphenyltltetrazolium bromide (MTT) viability assay is based on the ability of mitochondria to reduce MTT, a yellow tetrazolium dye, to MTT formazan, a blue mitochondrial byproduct. The reduction is mediated by mitochondrial dehydrogenases that are present in living but not dead cells, and the assay is appropriate to assess the viability of mitochondria‐rich cells. The myocytes were isolated as described here earlier and cultured in 200 μL of fresh DMEM/F12 medium supplemented with 20 μL of MTT (0.1 mg/mL) solution in a 96‐well plate (10^4^ cells/well). Four hours later, the supernatant was aspirated and 150 μL of DMSO was added to each well, and then the cells were incubated at 37°C for 10 minutes. The absorbance at 570 nm was then read for each well to determine myocyte viability (n=4).

### Protein Synthesis

Protein synthesis by myocytes was assessed by determining the incorporation of l‐[^14^C]phenylalanine (Phe; Amersham) into acid‐insoluble proteins. Myocytes were incubated with serum‐free DMEM/F12 supplemented with either unlabeled Phe 0.6 mmol/L, phenylephrine (PE) 100 μmol/L, or vehicle for 12, 24, and 48 hours, including 5 mCi/well l‐[^14^C]Phe for the last 4 hours. An excess of unlabeled Phe (0.6 mmol/L) was used to ensure the equilibration of intracellular and extracellular specific radioactivity. At the end of a 4‐hour labeling period, the cells were rapidly rinsed 3 times with ice‐cold PBS. Cells were solubilized in 2% sodium dodecyl sulfate and precipitated with 2 mL of 20% ice‐cold trichloroacetic acid (TCA). The precipitates were collected and washed sequentially with 10% and 5% TCA and finally with absolute ethanol. The radioactivity was determined in a Tri‐Carb 4000 liquid scintillation counter (Packard Instruments), and the [^14^C]Phe incorporation was presented as disintegrations per minute per l0^5^ cells. The cell protein synthesis rate was expressed as disintegrations per minute per well.

### Protein Degradation

To measure the rate of protein degradation, myocytes were labeled by preincubating cells in serum‐free DMEM containing 0.5 μCi/well l‐[^14^C]Phe for 48 hours. To minimize the reincorporation of l‐[^14^C]Phe, the experimental media contained an excess (2 mmol/L) of unlabeled Phe. After a 2‐hour phase, the cells were washed with PBS to remove l‐[^14^C]Phe released from the degradation of short‐lived proteins, and 4 mL of fresh experimental medium containing PE (100 μmol/L) was added. At the indicated sampling periods up to 48 hours, 0.3‐mL aliquots of the media were taken and precipitated by the addition of ice‐cold TCA (10% final concentration). The samples were centrifuged for 5 minutes, and the TCA‐soluble supernatant was frozen at −20°C overnight for the radioactivity measurement. At the end of each experiment, the cells were washed 3 times with ice‐cold PBS. The radioactivity in the cells and in the media was measured as described here earlier. The initial l‐[^14^C]Phe incorporation into cell proteins was calculated from the total radioactivity released plus the amount remaining within the cells at the end of the experiments. The protein degradation rate was calculated as the percentage of radioactivity released versus the radioactivity remaining in the cells at the indicated time intervals.

### Measurement of ATP

The ATP levels of myocytes or heart tissue from *Ctsl*^−/−^ and *Ctsl*^+/+^ mice were measured using the Enzylight ATP Assay kit (BioAssay Systems US), which denotes ATP bioluminescence as a marker of mitochondrial activity. Briefly, the phenothiazines were dissolved in PBS and dispensed in 10 μL aliquots into 96‐well microplates. The myocytes or fresh heart tissues were processed according to the manufacturer's protocol. The luminescence produced by the luciferase‐catalyzed luciferin plus ATP reaction was detected using a MicroLumat LB96P multiwall scanning spectrophotometer (EG&G Berthold). The data were then normalized to the control group. The percent decrease in ATP was calculated according to the decrease in luminescence compared with controls. Six independent experiments were carried out for each phenothiazine concentration, 1 experiment for each cell line, and 2 parallel samples for each phenothiazine concentration.

### Adeno‐Associated Virus Vectors and Gene Transfer

The full‐length human *CTSL1* gene was subcloned into the adeno‐associated virus 9 (AAV9)–green fluorescent protein (GFP) system.^17^ *CTSL1* was inserted upstream of the cytomegalovirus promoter into the AAV‐9 shuttle vector (Vector Biolabs). Control virus (AAV‐9 GFP alone) containing the cytomegalovirus‐GFP sequence only was custom‐made by Vector Biolabs. Neonatal ventricular myocytes were transfected with AAV9 constructs on the day of isolation. One hour after plating, AAV9‐*CTSL1* at 1×10^7^ viral genomes (VG) /well was added for 72 hours before further analysis.

### Animal Model

Transverse aortic banding (AB) was performed in 10‐week‐old male (25 to 27 g) CTSL‐deficient (B6×FSB/GnEia/a*Ctsl*^*fs*^/J; Jackson Laboratory) and wild‐type (*Ctsl*^*+/+*^) littermate control mice as described previously.^18^

#### Experimental Study 1

Animals were euthanized on postoperative weeks 2 and 8 (n=5 surviving animals per time point, per group). Hearts were harvested, rinsed with cold PBS, frozen, and stored at −80°C for immunohistochemical, biochemical, and histochemical studies.

#### Experimental Study 2

Cardiac function was assessed on postoperative weeks 2 and 8 (n=5 for sham per group, n=10 for AB per group) using echocardiography and on week 8 using LV pressure–volume loop acquisition (n=6 for sham per group, n=16 for AB per group). Animals were euthanized, and the hearts were perfusion‐fixed and then sectioned for cardiac morphometric studies.

### Immunohistochemistry

Cryostat sections (5 μm) were prepared, air‐dried, and fixed in 4% paraformaldehyde in PBS. The sections were incubated with 0.3% hydrogen peroxide and 10% BSA, followed by a primary antibody against CTSL (BD PharMingen Technical) overnight at 4°C and then incubation with a matching biotinylated secondary antibody (Vector) or fluorescent conjugated secondary antibody for 45 minutes at room temperature. Negative controls were performed for all immunologic staining by omitting the primary antibody.

### Isolation of Lysosomes and Measurement of Lysosomal Activity

Lysosomes were isolated using a lysosome isolation kit (Sigma) according to the manufacturer's instructions. Lysosomal proteins were quantified by a Bradford protein assay, and their intactness was measured. The dye absorbance of a neutral red dye at 460 and 510 nm was measured, and the neutral red uptake was calculated.

### LV Function

Eight weeks after the induction of AB, mice were anesthetized with ketamine (80 mg/kg) and xylazine (10 mg/kg) and placed on controlled heating pads. Core temperature was measured with a rectal probe and maintained at 37±0.5°C. A microtip catheter transducer (SPR‐839; Millar Instruments) was inserted into the right carotid artery and advanced into the left ventricle under pressure control. After 15 minutes of stabilization, the pressure and volume signals were recorded continuously with an ARIA pressure–volume conductance system coupled with a Powerlab/4SP A/D converter, stored, and displayed. PVAN software (Millar Instruments) was used for subsequent analysis of pressure–volume loops.

### Cardiac Morphometry

Morphometric analysis was performed on cardiac sections using an image quantitative digital analysis system (National Institutes of Health Image 1.6). Relative ventricular cavity dimension and wall thickness were determined according to the method of Sun et al.^19^ Single myocytes were measured with images captured from hematoxylin and eosin–stained sections. The outline of 100 to 200 myocytes was traced in each section. Simple PCR image system software (Universal Imaging) was used to determine myocyte cross‐sectional area.

### Transmission Electron Microscopy

The hearts were removed and immediately fixed in half‐strength Karnovsky's fixative as 3‐mm^3^ tissue cubes. The tissues were postfixed in 2% osmium tetroxide and embedded in araldite resin. Semithin sections were stained with toluidine blue/borax and examined by light microscopy. Ultrathin sections were stained with uranyl acetate and lead citrate and photographed using a Jeol 1200EX transmission electron microscope.^20^

### Lipid Peroxidation

Determination of malondialdehyde (MDA) was carried out to estimate the extent of lipid peroxidation in the heart. Tissue samples were added to butylated hydroxy toluene (1 mg/mL of Tris‐HCl 20 mmol/L), then frozen at −80°C until the assay was performed. After thawing, tissue samples were washed in ice‐cold Tris‐HCl 20 mmol/L, pH 7.4, minced in ice‐cold Tris‐HCl 20 mmol/L, and homogenized in a 1:10 weight/volume ratio with a Teflon pestle. After centrifugation at 3000*g* for 10 minutes at 4°C, the clear homogenate supernatant was used for the biochemical assay. The colorimetric commercial kit (lipid peroxidation assay kit; Calbiochem‐Novabiochem Corporation) was performed to assay free MDA. The concentration of MDA was normalized to sample protein content.

### Statistical Analysis

Statistical analyses were performed with GraphPad Prism 4.0. Survival between groups was compared by Kaplan–Meier survival analysis. All other comparisons were performed by nonparametric tests (Kruskal–Wallis test or Mann–Whitney test). The values are expressed as medians with 25th and 75th percentiles. Statistical significance is recognized at *P*<0.05.

## Results

### CTSL Activity Is Increased in Cardiac Myocytes With Hypertrophic Stimulation

**Figure 1.**
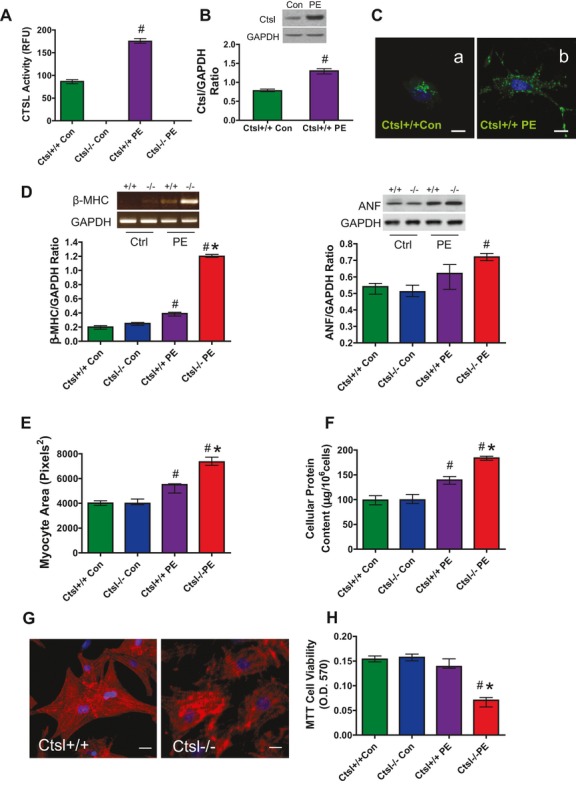
Isolated neonatal cardiac myocytes were treated with PE for 48 hours. A, CTSL activity measurements. B, Western blot detection and quantification of CTSL levels in *Ctsl*^+/+^ myocytes. C, Immunofluorescent staining for CTSL. D, Detection of β‐MHC mRNA by RT‐PCR and ANF protein by Western blotting. E, Myocyte area measurements. F, Cellular protein content. G, Immunofluorescent staining for α‐actinin. H, MTT assay for the cell viability. The values are expressed as medians with 25th and 75th percentiles (the bar indicates the median value and the line indicates the 25th and 75th percentiles; n=4). ^#^*P*<0.05 vs control, *P<0.05 vs *Ctsl*^+/+^ PE treatment. PE indicates phenylephrine; CTSL, cathepsin‐L; β‐MHC, beta‐myosin heavy chain; RT‐PCR, reverse transcription–polymerase chain reaction; ANF, atrial natriuretic factor; MTT, 3‐(4,5‐dimethylthiazol‐2‐yl)‐2.5‐diphenyltltetrazolium bromide.

PE stimulation led to an increase in the biological activity of CTSL in *Ctsl*^+/+^ myocytes compared with the control group without PE (Figure 1A). CTSL activity was not detected in *Ctsl*^−/−^ myocytes with or without PE stimulation. Furthermore, wild‐type myocytes exhibited low levels of CTSL at baseline (Figure 1B), mainly localized in the perinuclear region (Figure 1C‐a). In contrast, after stimulation with PE 100 μmol/L for 48 hours, CTSL level was increased and expressed diffusely throughout the cyoplasm as well as in the cell periphery (Figure 1B and 1C‐b).

### Hypertrophic Stimulation Results in an Exaggerated Cellular Response in *Ctsl*^−/−^ Myocytes

After PE stimulation, *Ctsl*^−/−^ neonatal cardiac myocytes exhibited increased hypertrophy as indicated by a significantly increased expression of fetal genes *ANF* and β‐*MHC* (Figure 1D), larger myocyte area (Figure 1E), and increased cellular protein content (Figure 1F). Immunofluorescent analysis of the *Ctsl*^−/−^ myocytes after PE stimulation for 48 hours demonstrated patchy aggregates and disorganization of the cytoskeletal protein α‐actinin compared with *Ctsl*^+/+^ myocytes (Figure 1G). These changes were also associated with decreased cell viability (Figure 1H). These results suggest that CTSL acts as a negative regulator of the cardiac hypertrophy process in response to PE stimulation.

### CTSL Is Required for Completing Autophagic Flux in Cardiac Myocytes With Hypertrophic Stimulation

**Figure 2.**
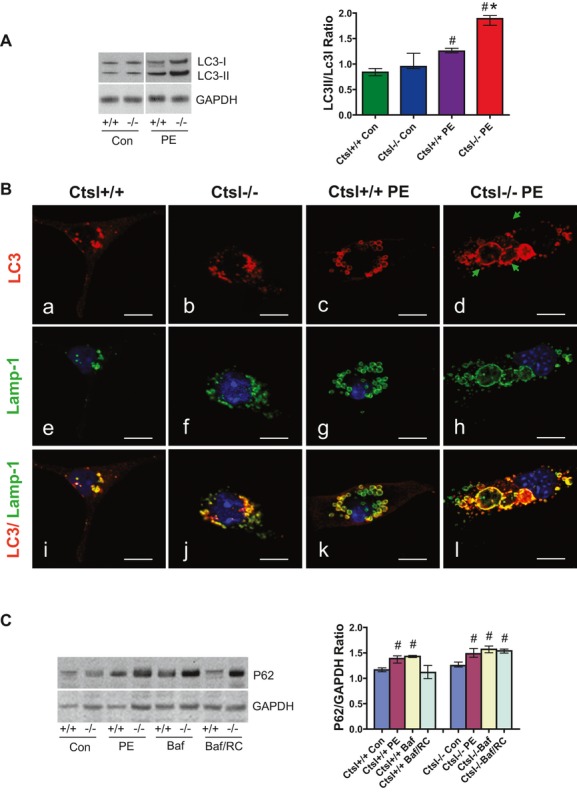
A, Representative Western blot and quantification of LC3 level after 48 hours of PE treatment. B, Immunofluorescent staining for LC3 (a through d), Lamp1 (e through h) and merged (i through l) in *Ctsl*^+/+^ (a, e, i) and *Ctsl*^−/−^ (b, f, j) control groups, and *Ctsl*^+/+^ (c, g, k) and *Ctsl*^−/−^ (d, h, i) after 48 hours of PE treatment. The arrows show the accumulation of large autophagosomes in PE‐treated *Ctsl*^−/−^ myocytes. C, Western blot detection and quantification of p62 levels in *Ctsl*^+/+^ and *Ctsl*^−/−^ myocytes treated with PE, bafilomycin A1 (Baf), and Baf recovery (Baf/RC). Values are expressed as medians with 25th and 75th percentiles (n=4). ^#^*P*<0.05 vs control, *P<0.05 vs *Ctsl*^+/+^ PE treatment. LC3 indicates microtubule‐associated protein 1 light chain 3; PE; phenylephrine; CTSL, cathepsin‐L.

Levels of LC3‐II, an autophagosome activation marker,^21^ were increased in *Ctsl*^*−/−*^ myocytes after 48 hours of PE treatment compared with *Ctsl*^*+/+*^ controls (Figure 2A). This was accompanied by increased accumulation of LC3‐positive vesicles on immunofluorescence analysis (Figure 2B). Clearance of autophagosomes occurs via fusion with lysosomes, followed by the degradation of autophagolysosomal content. To determine whether CTSL deficiency affected autophagosome–lysosome fusion and degradation, we analyzed LC3‐II and lysosomal membrane protein (Lamp1) colocalization in myocytes before and after PE treatment. Under basal conditions, LC3‐II and Lamp colocalized in *Ctsl*^*+/+*^ myocytes, and this pattern of staining was similar in the *Ctsl*^*−/−*^ myocytes (Figure 2B‐i, j). After PE stimulation, the LC3‐positive autophagosomes and Lamp1‐positive lysosomes showed an increase in both groups (Figure 2B‐k, l), but this was more pronounced in *Ctsl*^*−/−*^ myocytes. However, the colocalization pattern did not differ significantly between the 2 groups. These data suggest that the formation of autophagosomes and the fusion of autophagosome with lysosomes were not impaired by CTSL deficiency following hypertrophic stimulation. Accumulation of autophagosomes was likely due to defective clearance caused by lack of CTSL, leading to impaired lysosomal activity.

In addition, impaired autophagy–lysosomal degradation in PE‐treated *Ctsl*^*−/−*^ cardiac myocytes is reflected by the significantly increased level of p62, an adapter protein critical for bridging ubiquitinated protein to autophagosomes (Figure 2C). To further validate that deficiency of CTSL caused an impairment of autophagic activity and degradation, we monitored autophagic flux using bafilomycin A1 (Baf), a lysosomal inhibitor, in *Ctsl*^*+/+*^ and *Ctsl*^*−/−*^ myocytes. The results showed that p62 levels were increased in both PE‐treated and nontreated cardiac myocytes. However, in *Ctsl*^*+/+*^, the level of p62 showed further enhancement on Baf treatment, which reduced to the normal level after Baf removal. However, p62 level remained high in *Ctsl*^*−/−*^ myocytes despite the removal of Baf. In the absence of CTSL, extra‐large autophagosomes accumulated in the myocytes, which further exaggerated the hypertrophy response (Figure 2B‐d). These findings indicate impairment of lysosomal degradation and retardation of autophagic flux in *Ctsl*^*−/−*^ due to deficiency of CTSL.

### Decreased Protein Processing and Turnover in *Ctsl*^−/−^ Myocytes With PE Stimulation

**Figure 3.**
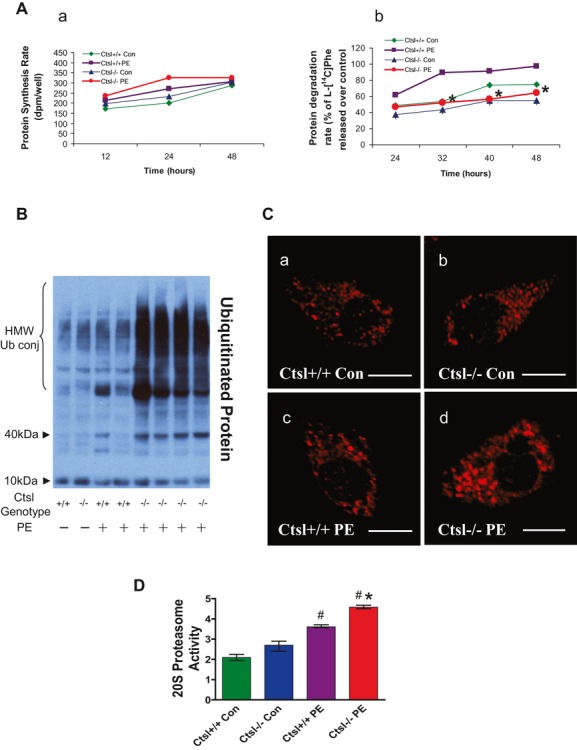
A, Effect of phenylephrine (PE) on protein synthesis (a) and degradation (b) in *Ctsl*^+/+^ and *Ctsl*^−/−^ neonatal myocytes (n=5); *P<0.05 Ctsl^+/+^ vs *Ctsl*^−/−^ groups. B, Western blot of ubiquitin protein in *Ctsl*^+/+^ and *Ctsl*^−/−^ neonatal myocytes following PE treatment. C, Immunofluorecent detection of ubiquitin protein in *Ctsl*^+/+^ (a, c) and *Ctsl*^−/−^ (b, d) following control (a, b) or PE treament (c, d) in neonatal myocytes. D, Measurement of 20S proteasome activity in *Ctsl*^+/+^ and *Ctsl*^−/−^ neonatal myocytes following PE treatment (n=4). ^#^*P*<0.05 vs control, *P<0.05 vs *Ctsl*^+/+^ PE treatment. Values are expressed as medians with 25th and 75th percentiles. PE indicates phenylephrine; CTSL, cathepsin‐L; HMW, high molecular weight.

The autophagy–lysosomal pathway plays an important role in cellular protein turnover, particularly under stress. Cardiac myocyte protein synthesis and degradation were investigated in cells exposed to PE (100 μmol/L). As seen in Figure 3A, PE stimulated the incorporation of l‐[^14^C]Phe into cells in a time‐dependent manner (up to 48 hours) in both PE‐treated *Ctsl*^*+/+*^ and *Ctsl*^*−/−*^ myocytes. This shows no significant difference in incorporation (Figure 3A‐a) between groups. However, the rates of protein degradation, as reflected by l‐[^14^C] Phe release, were significantly decreased in *Ctsl*^*−/−*^ compared with *Ctsl*^*+/+*^ PE‐treated myocytes between time periods of 32 to 48 hours (Figure 3A‐b).

### Effect on Ubiquitin‐Proteasome in *Ctsl*^−/−^ Myocytes After Hypertrophic Stimulation

UPS and selective autophagy are 2 major pathways regulating proteolysis and protein quality control. A defect in either system could result in ubiquitinated protein aggregated as seen in the CTSL‐deficient myocytes. In this study, the ubiquitinated proteins were significantly accumulated in CTSL‐deficient myocytes, especially after PE stimulation (Figure 3B). The immunostaining detected the ubiquitin‐positive protein distribution in both the cytoplasm and nucleus of stimulated *Ctsl*^−/−^ myocytes (Figure 3C). This raises the possibility that deficiency of CTSL impairs the clearance of misfolded proteins and results in the accumulation of ubiquitinated protein aggregates. However, it could also be the result of increased activation of the UPS pathway due to the defect in autophagy–lysosomal degradation pathway. Therefore, 20S proteasomal activity was measured. As seen in Figure 3D, there were significantly high levels in both control and PE‐treated myocytes in *Ctsl*^−/−^ compared with *Ctsl*^*+/+*^. Therefore, the increased UPS activity in CTSL deficiency is a compensatory mechanism to overcome the impairment of autophagy–lysosomal degradation.

### Restoration of Protein Processing by AAV9‐Mediated *CTSL1* Transfer In Vitro

**Figure 4.**
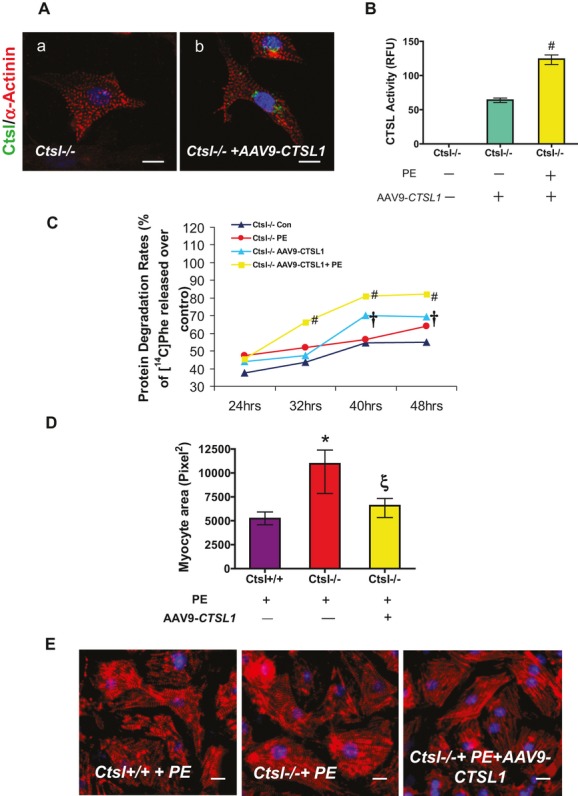
AAV9‐mediated *CTSL1* transfection in CTSL‐deficient myocytes. A, Immunofluorecent detection of *CTSL1* expression in *Ctsl*^−/−^ neonatal myocytes after AAV9‐mediated *CTSL1* gene transfer (a, control; b, AAV9‐*CTSL1* transgenic). B, CTSL activity in control and gene transfer *Ctsl*^−/−^ myocytes with and without PE treatment (n=4). ^#^*P*<0.05 vs *CTSL1* gene transferred without PE treatment myocytes. C, Effect of PE (100 μmol/L) on protein degradation in *Ctsl*^−/−^ and AAV9 mediated *CTSL1* gene transferred *Ctsl*^−/−^ neonatal myocytes (n=5). ^†^*P*<0.05 vs *Ctsl*^−/−^ control. ^#^*P*<0.05 vs *Ctsl*^−/−^ PE treatment. D, Myocyte area measurements in PE‐treated *Ctsl*^+/+^, *Ctsl*^−/−^ and *Ctsl*^−/−^ AAV9‐mediated *CTSL1* gene transferred myocytes. ^**ξ**^*P*<0.05 vs *Ctsl*^−/−^ non‐AAV9‐*CTSL1* gene transferred PE treated group, *P<0.05 vs *Ctsl*^+/+^ non‐AAV9‐*CTSL1* gene transferred PE treatment. E, Immunofluorescent staining for cardiac α‐actinin in PE treated *Ctsl*^+/+^, *Ctsl*^−/−^ and *Ctsl*^−/−^ AAV9 mediated *CTSL1* gene transferred myocytes. Values are expressed as medians with 25th and 75th percentiles. AAV9 indicates adeno‐associated virus 9; . PE, phenylephrine; CTSL, cathepsin‐L; CX4.

To evaluate further the specificity of CTSL on protein processing and determine the potential therapeutic value of CTSL gain of function, we expressed human *CTSL1* through an AAV9 viral delivery system in *Ctsl*^−/−^ myocytes. Immunostaining using human *CTSL1* antibody detected the *CTSL1* expression in transfected *Ctsl*^−/−^ myocyes (Figure 4A). The CTSL activity assay showed potential recovery of CTSL activity in the *Ctsl*^−/−^ AAV9‐*CTSL1*–transduced myocytes and was further upregulated by PE treatment (Figure 4B). Moreover, the impairment in protein turnover caused by CTSL deficiency was partially rescued. As shown in Figure 4C, protein degradation rate was significantly increased in *CTSL1* gene–transduced myocytes. Myocyte size was also significantly reduced compared with the nontransduced group (Figure 4D), together with improved α‐actinin organization (Figure 4E).

### Increased Cardiac CTSL Expression and Activity After AB

**Figure 5.**
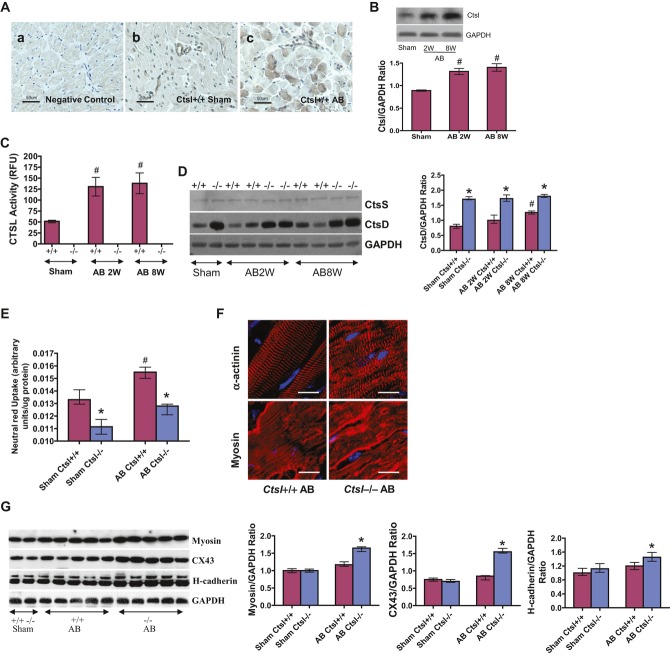
A, Immunohistochemical detection of CTSL expression in the heart of *Ctsl*^+/+^ mice. Negative control (a), sham surgery (b) and AB (c). CTSL was expressed in myocytes, endothelial, smooth muscle, and fibroblast cells in sham mice hearts. After AB, CTSL expression was increased in cardiomyocytes. B, Western blot detection and the quantification of CTSL level in *Ctsl*^+/+^ sham and banded heart (n=5). C, Representative CTSL activity following AB in *Ctsl*^+/+^ and *Ctsl*^−/−^ hearts (n=5). D, Western blot of CtsS and CtsD protein levels and quantification data in *Ctsl*^+/+^ and *Ctsl*^−/−^ mice hearts after sham and AB surgery. E, Lysosomal neutral red uptake in *Ctsl*^+/+^ and *Ctsl*^−/−^ mice hearts after sham and AB surgery (n=4); ^#^*P*<0.05 vs sham group; *P<0.05 vs *Ctsl*^+/+^ groups. F, Immunofluorescent staining for cardiac α‐actinin and myosin in *Ctsl*^+/+^ and *Ctsl*^−/−^ AB mice. G, Western blot of myosin, CX43, and H‐cadherin proteins and quantification data in the heart tissues from *Ctsl*^+/+^ and *Ctsl*^−/−^ mice (n=5); ^#^*P*<0.05 vs sham group; *P<0.05 vs *Ctsl*^+/+^ groups. Values are expressed as medians with 25th and 75th percentiles. RFU indicates relative fluorescence units; CTSL, cathepsin‐L; AB, aortic banding; CX43, connexin 43.

To evaluate the role of CTSL in cardiac remodeling in vivo, mice were subjected to AB. As shown in Figure 5A, cardiac CTSL expression was detected by immunohistochemistry in myocytes, endothelial cells, smooth muscle cells, and fibroblasts. After AB, CTSL expression was increased in all cell types, especially in cardiomyocytes. CTSL protein levels were also confirmed by Western blotting (Figure 5B). High levels of CTSL activity were also maintained to 8 weeks post AB in *Ctsl*^+/+^ mice but were not detectable in *Ctsl*^−/−^ mice (Figure 5C).

### Changes in Cathepsin Family Members in Hearts Subjected to AB

To determine whether CTSL deficiency induced compensatory changes in other members of the cathepsin family, we evaluated cathepsin‐S and ‐D by Western blot analysis. As shown in Figure 5D, there was no change in cathepsin‐S levels with or without AB in both groups. However, increased levels of cathepsin‐D were observed at baseline in *Ctsl*^−/−^ compared with *Ctsl*^+/+^ mice. After AB, cathepsin‐D levels were increased in *Ctsl*^+/+^ mice but did not increase further in *Ctsl*^−/−^ mice.

### CTSL Deficiency Impaired Lysosomal Activity, Leading to Myofilament Disorganization and Aggregate Formation in Response to AB

As expected, there was reduced lysosomal neutral red uptake, indicating impaired lysosomal function^22–24^ in *Ctsl*^−/−^ mice compared with *Ctsl*^+/+^ controls (Figure 5E). In addition, although lysosomal activity was upregulated post AB, the relative increase in neutral red uptake is still significantly lower in the *Ctsl*^*−/−*^ compared with the *Ctsl*^+/+^ group (Figure 5E). Confocal microscopy and immunohistochemical analyses of heart sections revealed an orderly arrangement of cytoskeletal proteins in *Ctsl*^+/+^ cardiac myocytes post AB. In contrast, cytoskeletal proteins α‐actinin and myosin were disorganized and aggregated in clumps in *Ctsl*^*−/−*^ AB mice (Figure 5F). Western blot analysis and quantification demonstrated increased levels of myosin, Connexin43, and H‐cadherin in *Ctsl*^−/−^ hearts compared with *Ctsl*^+/+^ mice (Figure 5G) after AB.

### CTSL Deficiency Worsened Ventricular Remodeling and Heart Failure in Response to AB

**Figure 6.**
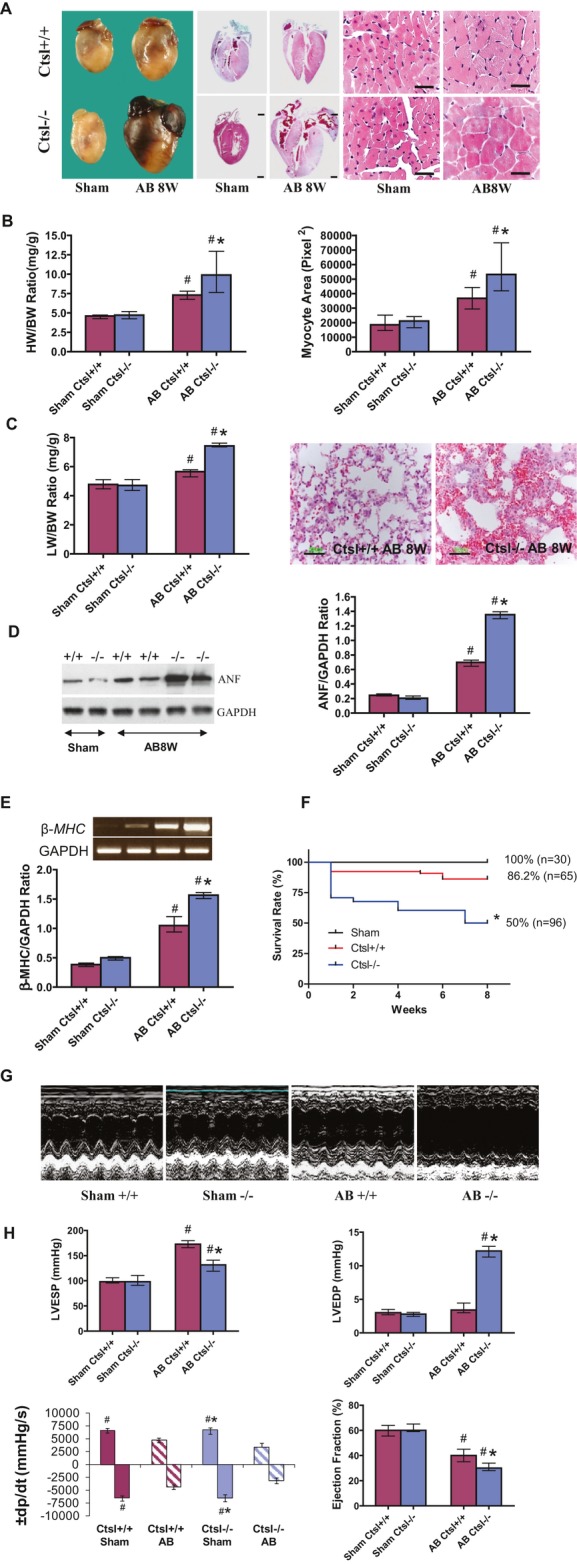
A, Representative gross morphology of whole hearts, corresponding coronal sections (scale bar, 2 mm), and H&E staining of hearts 8 weeks post sham and AB surgery. B, Quantitative data of heart weight–to–body weight ratio and myocyte area (n=16). C, Lung‐to‐body weight ratio (n=16) and H& E staining of lung tissue 8 weeks post AB. D, Western blot detection and the quantification of cardiac ANF levels (n=4). E, RT‐PCR detection for β‐MHC mRNA expression (n=4). B through E: ^#^*P*<0.05 vs sham group; *P<0.05 vs *Ctsl*^+/+^ AB groups. F, Kaplan–Meier survival curves in *Ctsl*^+/+^ (n=65) and *Ctsl*^−/−^ (n=96) mice after AB or sham‐operation (n=30/genotype). *P<0.001 vs *Ctsl*^−/−^. G, Representative M‐mode echocardiograms of *Ctsl*^+/+^ and *Ctsl*^−/−^ mice 8 weeks post sham and AB surgery. H, In vivo hemodynamic measurements: left ventricular end‐systolic pressure (LVESP), LV end‐diastolic pressure (LVEDP), peak rates of pressure rise (+dP/dt) and pressure fall (−dP/dt), and ejection fraction (EF) 8 weeks post sham surgery (n=6) or AB (n=16) in *Ctsl*^+/+^ and *Ctsl*^−/−^ mice. ^#^*P*<0.05 vs sham group; *P<0.05 vs *Ctsl*^+/+^ AB groups. Values are expressed as medians with 25th and 75th percentiles. CTSL indicates cathepsin‐L; AB, aortic banding; ANF, atrial natriuretic factor; H&E, hematoxylin and eosin; β‐MHC, beta‐myosin heavy chain; RT‐PCR, reverse transcription–polymerase chain reaction.

AB resulted in cardiac hypertrophy in both *Ctsl*^+/+^ and *Ctsl*^−/−^ mice within 2 weeks but was exaggerated in *Ctsl*^−/−^ mice (Figure 6A). As expected, the heart weight–to–body weight ratio of *Ctsl*^+/+^ mice increased by 36%, and the size of LV myocytes increased by 33% post AB. In comparison, the heart weight–to–body weight ratio was increased by 78% and LV myocyte size increased by 72% (*P*<0.01) in *Ctsl*^−/−^ mice post AB (Figure 6B). Lung weight–to–body weight ratio was also significantly increased post AB in both groups, especially in *Ctsl*^−/−^ mice (Figure 6C). The lungs of *Ctsl*^*+/+*^ mice showed mild edema and cellular infiltration in the alveolae post AB (Figure 6C). On the other hand, the alveolar septa appeared thickened and the alveolar space contained numerous cells and edema in *Ctsl*^−/−^ mice (Figure 6C). More robust increases in ANF protein level and β‐MHC mRNA expression were observed in *Ctsl*^−/−^ mice, with high levels persisting at 8 weeks post AB (Figure 6D and 6E).

### CTSL Deficiency Worsened Systolic and Diastolic Function and Survival Rate in Response to AB

In our study, AB generated comparable pressure gradients immediately post surgery (42 to 48 mm Hg) independent of genotype (data not shown), but there was a significantly lower survival rate in the *Ctsl*^−/−^ mice compared with the *Ctsl*^+/+^ mice (Figure 6F). Survival rate at 8 weeks was 86% and 50% for *Ctsl*^+/+^ and *Ctsl*^−/−^ mice, respectively (*P*<0.001).

M‐mode echocardiography revealed exacerbated LV dilatation in response to AB in *Ctsl*^−/−^ mice (Figure 6G). As early as 2 weeks post AB, the thickness of the interventricular septum and the LV posterior wall, as well as LV end‐systolic and ‐diastolic diameters, were all increased compared with *Ctsl*^+/+^ mice (Table 1). Cardiac dysfunction was further confirmed by in vivo hemodynamic measurements. *Ctsl*^−/−^ AB mice had lower LV end‐systolic pressure, higher LV end‐diastolic pressure, and markedly depressed LV systolic and diastolic dP/dt values compared with *Ctsl*^+/+^ AB mice (Figure 6H). Reduced –dP/dt and increased LV end‐diastolic pressure suggested diastolic dysfunction in *Ctsl*^−/−^ mice post AB (Figure 6H).

**Table 1.** M‐Mode Echocardiography Data 2 and 8 Weeks Post AB

	*Ctsl* ^+/+^			*Ctsl* ^−/−^		
	Sham	AB		Sham	AB	
		2 wk	8 wk		2 wk	8 wk
LVEDD, mm						
Median	3.57	4.15	4.5*	3.62	4.7*	6.14**
(25% to 75%)	3.28 to 3.73	3.89 to 4.28	4.2 to 5.0	3.31 to 3.7	4.3 to 4.86	5.83 to 6.37
LVESD, mm						
Median	2.09	2.59*	2.82*	2.11	3.24**	4.57**
(25% to 75%)	1.73 to 2.33	2.47 to 2.71	2.48 to 3.48	1.79 to 2.51	3.05 to 3.3	4.4 to 5.15
IVSd, mm						
Median	0.53	0.66	0.78*	0.61	0.87**	0.92**
(25% to 75%)	0.50 to 0.66	0.59 to 0.72	0.71 to 0.87	0.58 to 0.66	0.81 to 0.92	0.87 to 0.98
PWd, mm						
Median	0.58	0.71	0.89*	0.59	0.87*	1.27**
(25% to 75%)	0.55 to 0.63	0.66 to 0.77	0.82 to 0.99	0.56 to 0.68	0.8 to 0.97	1.12 to 1.39
% FS						
Median	42.4	37.3	36.5*	39.55	28.15*	21.7*
(25% to 75%)	33.9 to 45.7	34 to 40	26 to 42	31.5 to 47.5	26.2 to 29.5	1.12 to 1.39
HR, /min						
Median	532	542	494	554	558	542
(25% to 75%)	460 to 560	487 to 556	429 to 543	521 to 566	507 to 579	453 to 573
N	5	10	10	5	10	10

Two‐dimensionally guided M‐mode echocardiography and hemodynamics were studied at 2 and 8 weeks post AB. AB indicates aortic banding; Ctsl, cathepsin‐L; LVEDD, left ventricular end‐diastolic diameter; LVESD, left ventricular end‐systolic diameter; IVSd, diastolic interventricular septal thickness; PWd, diastolic posterior wall thickness; FS, fractional shortening; HR, heart rate; N, animal number.

* *P*<0.05 vs sham group.

* *P*<0.05 vs *Ctsl*^+/+^ AB groups.

### CTSL Deficiency Causes Increased Hsp70 and Ubiquitinated Proteins in Response to AB

**Figure 7.**
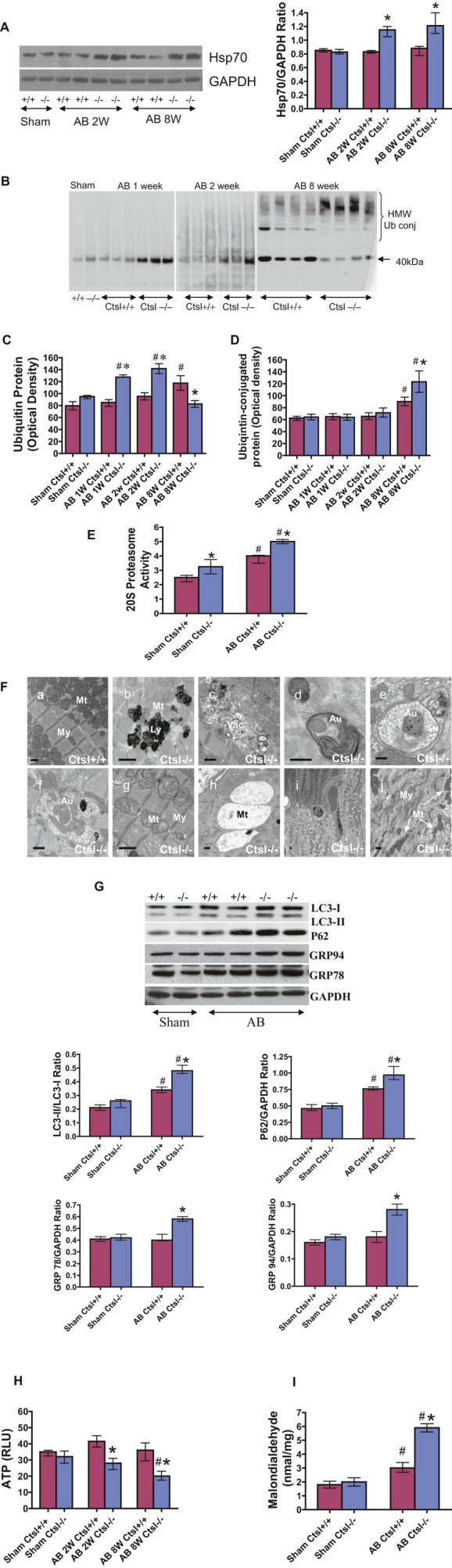
A, Western blot for Hsp70 and the quantification in *Ctsl*^+/+^ and *Ctsl*^−/−^ mice after sham and AB surgery. B, Western blot for cardiac ubiquitin protein in *Ctsl*^+/+^ and *Ctsl*^−/−^ mice after 1, 2, and 8 weeks post sham and AB surgery. C and D, Quantitative results of cardiac ubiquitin protein in (B). E, 20S proteasome activity measurement. F, Electron microscopy of myocardium at 2 weeks post AB. CTSL deficient mice demonstrated end‐stage lysosomes, vacuolation, autophagosome formation, and mitochondrial degeneration (Mt, mitochondria; My, myofibrils; Ly, lysosome; Vac, vacuoles; Au, autophagosome). Scale bar, 500 nm. G, Western blot of LC3, p62, GRP78 and GRP94 protein and the quantification in the heart tissue from *Ctsl*^+/+^ and *Ctsl*^−/−^ mice. H, ATP production. I, Increased malondialdehyde levels in *Ctsl*^−/−^ mice post AB (n=4 to 6). ^#^*P*<0.05 vs sham; *P<0.05 vs *Ctsl*^+/+^ AB groups. Values are expressed as medians with 25th and 75th percentiles. CTSL indicates cathepsin‐L; AB, aortic banding; GRP, green fluorescent protein; Hsp70, heat shock protein 70.

Hsps, including Hsp70, are molecular chaperones that attempt to refold misfolded proteins and prevent the accumulation of cytoplasmic protein aggregates. In the present study, Hsp70 levels were significantly increased in banded *Ctsl*^−/−^ mice (Figure 7A). In *Ctsl*^*+/+*^ mice, the 40‐kDa ubiquitin protein levels were increased significantly after 8 weeks of AB (Figure 7B and 7C). In contrast, the 40‐kDa ubiquitin protein levels in *Ctsl*^−/−^ mice were significantly increased at 1 and 2 weeks and decreased dramatically after 8 weeks (Figure 7B and 7C). This decrease in 40‐kDa ubiquitin was accompanied by an increase in high molecular weight conjugated ubiquitin, depicted in Figure 7B. Densitometry revealed a 92.4% and 44.9% increase in conjugated ubiquitin protein levels after 8 weeks of AB in *Ctsl*^−/−^ and *Ctsl*^+/+^ mice, respectively, compared with sham operated mice(Figure 7D). S20 proteasome activity analysis presented a significantly high level in both sham and AB conditions compared with *Ctsl*^+/+^ mice (Figure 7E). Therefore, the increased levels of ubiquitinated proteins in *Ctsl*^−/−^ hearts following AB suggest a possibility of compensatory mechanism to overcome the impairment of autophagy–lysosomal pathway.

### Ultrastructural and Biochemical Alterations in CTSL‐Deficient Mice in Response to AB

The tissue ultrastructure of *Ctsl*^*+/+*^ AB hearts showed little vacuolation and an absence of abnormal lysosomal accumulation (Figure 7F‐a). In contrast, an aberrant myocardial ultrastructure was observed in *Ctsl*^−/−^ AB myocardium, characterized by the accumulation of numerous end‐stage lysosomes with high electron density deposits (Figure 7F‐b), accompanied by a robust accumulation of vacuolation (Figure 7F‐c) and autophagosomes (Figure 7F‐d through f). Furthermore, swelling and degeneration of the mitochondrial matrix (Figure 7F‐g, h, i) with disorganized myocyte filament fibers (Figure 7F‐j) were observed in these myocytes. An increased ratio of LC3‐II/LC3‐I and p62 levels in *Ctsl*^*−/−*^ mice demonstrated an impairment of autophagy–lysosomal response to pressure overload (Figure 7G), which were confirmed in our in vitro studies. Western blot analysis showed increased levels of GRP78 and GRP94 in *Ctsl*^−/−^ mice post AB (Figure 7G), suggesting that these 2 stress proteins work in concert during the folding and assembling of proteins.^25^ Neither protein nor ultrastructural changes were apparent in the myocardium of *Ctsl*^*+/+*^ AB cardiac myocytes.

*Ctsl*^−/−^ mice showed high mortality after AB due to impaired cardiac contractility and heart failure. As shown in Figure 7H, ATP productions were similar in sham groups but were reduced at 2 weeks and even further at 8 weeks post AB in *Ctsl*^−/−^ mice compared with *Ctsl*^*+/+*^ mice. Finally, a greater degree of oxidative stress, as measured by MDA, was observed in *Ctsl*^−/−^mice compared with *Ctsl*^*+/+*^ post AB (Figure 7I).

## Discussion

Growing evidence suggests that impairment of the lysosomal system may lead to the accumulation of abnormal proteins in severe cardiomyopathy and aging.^12–13,12–27^ These observations prompted us to examine the lysosomal protease CTSL as a modulator of protein turnover. Our current study demonstrates that CTSL plays an important role in protecting against cardiac dysfunction following pressure overload via activation of the autophagy–lysosomal pathway coordinated with the UPS, thus regulating protein turnover and cellular homeostasis.

Pathological cardiac hypertrophy is a major predictor of heart failure, which is itself associated with a mortality of 25% to 30% at 1 year.^28–29^ Despite the importance of hypertrophy as a prelude to heart failure, mechanisms governing this transition remain poorly understood. Autophagy has recently been recognized to play a critical role in the regulation of cardiac remodeling in response to stress.^30–31^ Increases in autophagy have been observed in cardiac disease^31^ and have been correlated with accelerated cell death and heart failure.^30^

CTSL is thought to participate in the lysosomal proteolytic processes, a role supported by its endosomal and lysosomal localizations, as well as its redundant substrate specificities within the heart. In support of this theory, in vitro study found that hypertrophic stimulation with PE led to a significantly increase in cellular CTSL expression and activity (Figure 1). These alterations may be part of the cell's compensatory response to maintain intracellular homeostasis in response to hypertrophic stimuli. CTSL deficiency led to reduction of lysosomal activity, with accumulation of autophagosomes, and evidence of degradation of the autophagolysosomal content is impaired (Figure 2). A considerable accumulation of ubiquitinated proteins was observed in CTSL‐deficient myocytes in response to PE, compensated with increased UPS activity, suggesting the coordinated involvement of the UPS. Furthermore, CTSL‐deficient myocytes showed significant hypertrophic changes with aggregation of cellular filaments, as well as a decrease in cellular protein degradation rate and cell viability (Figure 3). These observations were confirmed with the partial rescue of the *Ctsl*^−/−^ protein degradation rate and hypertrophic responses by the AAV9‐mediated human *CTSL1* gene transfer (Figure 4). These data suggested that an alteration of the lysosomal activity by CTSL deficiency resulted in impairment of protein processing.

The in vivo murine model of pressure overload–induced hypertrophy furthered our understanding of the role of CTSL in mammalian cardiac remodeling. Consistent with the results of in vitro study, pressure overload induced robust expression and activation of CTSL in the hearts of wild‐type mice. In contrast, CTSL‐deficient mice had a compensated upregulation of cathepsin‐D levels, thus maintaining some degree of basal lysosomal function under physiological conditions without major impairment in cellular function. However, under the stress of pathological hypertrophy, cathepsin‐D levels failed to further elevate and were unable to compensate for the CTSL deficiency. Impaired lysosomal function (Figure 5) may result in the accumulation of autophagosomes and insufficiently digested proteins. The alterations of sarcomeric and cytoskeletal components observed in this study may account for the impaired generation and transmission of cardiac force in these mice.^13^ The rapid and significant increase in cardiac mass and features of heart failure in *Ctsl*^−/−^ mice under pressure overload indicated that CTSL plays a critical role in the response to pathological stress by maintaining protein homeostasis.^32^

Intracellular protein turnover and protein quality control are regulated through lysosomal proteolysis and the UPS^1^ and may partially compensate for each other.^33–36^ The proteolytic function of the UPS often becomes inadequate in proteinopathies, which in turn leads to activation of autophagy to remove abnormal protein, especially those in aggregated forms. Impairment of the autophagy–lysosomal pathway due to CTSL deficiency caused an increase in protein ubiquitination by the UPS. However, these proteins aggregated in the cardiac myocyte (Figure 7) and were unable to be cleared.^37^ Therefore, integration of the UPS and autophagy systems seems to be dependent on the integrity of lysosomal function.^35^ Dysregulated autophagy may contribute to the pathogenesis of heart disease,^38–39^ including heart failure.^37,40^ We have shown here that an early increase in autophagic vacuole formation is observed in *Ctsl*^−/−^ hearts after AB. This lack of CTSL led to decreased lysosomal proteolytic activities, impaired lysosomal fusion and/or autophagosomal content degradation, autophagosome accumulation, and mitochondrial dysfunction, ultimately leading to cellular dysfunction and loss.^41–45^

Intracellular accumulation of proteins may disrupt homeostasis and lead to organelle toxicity. Mitochondria, which continually undergo remodeling, are the major source of ATP in the heart and are tightly associated with mechanical performance.^46–47^ In *Ctsl*^−/−^ AB mice, we observed a decrease in cellular ATP along with abnormal mitochondrial structures, suggesting a functional link between the 2. Abnormal mitochondrial accumulation can lead to the release of proapoptotic factors, which enhances oxidative stress and leads to protein oxidation, DNA breaks, and lipid peroxidation.^35^ As increased reactive oxygen species generation was observed in the hearts of CTSL‐null mice,^12^ we have also shown that its activation is further increased after AB, which may be the result of disrupted endoplasmic reticulum (ER) homeostasis.^39^ Post AB, ER molecular chaperones BiP/GRP78 and GRP94, as well as the non‐ER chaperone HSP70, were elevated in *Ctsl*^−/−^ mice. These findings suggest CTSL deficiency leads to ER stress that contributes to cardiac dysfunction and cellular apoptosis. Together, these data indicate a compensatory chaperone‐mediated mechanism for the defective lysosomal function observed in CTSL‐deficient mice (Figure 7).

**Figure 8.**
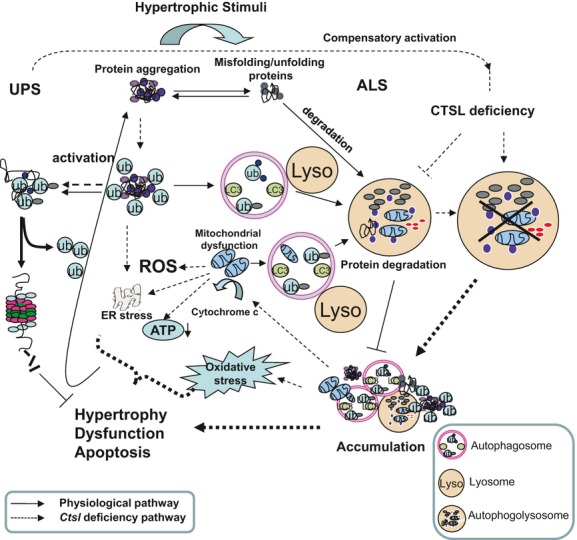
Protein quality control and autophagy–lysosomal systems (ALS). Myocyte proteins are normally turned over by the activities of the ALS and the ubiquitin–proteasome system (UPS). In CTSL‐deficient myocyte, an accelerated response to pathological stress represents a basis for pathogenic protein accumulations, resulting in exacerbated cardiac hypertrophy, cell dysfunction, and death. This was associated with the defective lysosomal function, leading to an impairment of the autophagy–lysosomal pathway, and ultimately resulting in impaired protein degradation and intracellular homeostatic remodeling. CTSL indicates cathepsin‐L; ROS, reactive oxygen species; ER, endoplasmic reticulum; ub, ubiquitin.

In conclusion, mice with CTSL deficiency demonstrated an accelerated response to pathological stress resulting in exacerbated cardiac hypertrophy and dysfunction. This was associated with the defective lysosomal function, leading to an impairment of the autophagy–lysosomal pathway, and ultimately resulting in impaired protein degradation and intracellular homeostatic remodeling (Figure 8). By maintaining lysosomal function and ensuring adequate protein quality control, CTSL plays an integral role in the remodeling response of the heart to stress.

## Acknowledgment

We thank Xin Chen and Liyong Zhang for preparing reagents for revision experiments.
